# Assessment of the competence in electrocardiographic interpretation among Arabic resident doctors at the emergency medicine and internal medicine departments: A multi-center online cross-sectional study

**DOI:** 10.3389/fmed.2023.1140806

**Published:** 2023-04-24

**Authors:** Amine Rakab, Sarya Swed, Hidar Alibrahim, Haidara Bohsas, Yasmeen Abouainain, Kirellos Said Abbas, Yazan Khair Eldien Jabban, Bisher Sawaf, Bushra Rageh, Majd Alkhawaldeh, Israa Al-Fayyadh, Mohamad Saad Rakab, Sherihan Fathey, Wael Hafez, Amr Gerbil, Emad Hassan Hassan El-Shafei

**Affiliations:** ^1^Clinical Medicine, Weill Cornell Medical College, Doha, Qatar; ^2^Faculty of Medicine, Aleppo University, Aleppo, Syria; ^3^Faculty of Medicine, University of Jordan, Amman, Jordan; ^4^Faculty of Medicine, Alexandria University, Alexandria, Egypt; ^5^Faculty of Medicine, Damascus University, Damascus, Syria; ^6^Department of Internal Medicine, Hamad Medical Corporation, Doha, Qatar; ^7^Faculty of Medicine, Sanaa University, Sanaa, Yemen; ^8^Faculty of Medicine, Mansoura University, Mansoura, Egypt; ^9^Ministry of Health, New Giza University, Giza, Egypt; ^10^NMC Royal Hospital, Abu Dhabi, United Arab Emirates; ^11^Medical Research Division, Department of Internal Medicine, The National Research Center, Cairo, Egypt; ^12^Critical Care Medicine, Mediclinic Alnoor Hospital, Abu Dhabi, United Arab Emirates

**Keywords:** electrocardiographic, emergency medicine, internal medicine, multi-center cross sectional, Middle East

## Abstract

**Background:**

This study aims to assess the electrocardiographic interpretation abilities of resident doctors at internal medicine and emergency medicine departments in eight Arabic countries.

**Methods:**

An online cross-sectional study was conducted between October 7, 2022 and October 21, 2022 in eight Arabic countries. The questionnaire consisted of two main sections: the first section included sociodemographic information, while the second section contained 12 clinical case questions of the most severe cardiac abnormalities with their electrocardiography (ECG) recordings.

**Results:**

Out of 2,509 responses, 630 were eligible for the data analysis. More than half of the participants were males (52.4%). Internal medicine residents were (*n* = 530, 84.1%), whereas emergency medicine residents were (*n* = 100, 15.9%). Almost participants were in their first or second years of residency (79.8%). Only 36.2% of the inquired resident doctors had attended an ECG course. Most participants, 85.6%, recognized the ECG wave order correctly, and 50.5% of the participants scored above 7.5/10 on the ECG interpretation scale. The proportions of participants who were properly diagnosed with atrial fibrillation, third-degree heart block, and atrial tachycardia were 71.1, 76.7, and 56.6%, respectively. No statistically significant difference was defined between the internal and emergency medicine residents regarding their knowledge of ECG interpretation (*p* value = 0.42). However, there was a significant correlation between ECG interpretation and medical residency year (*p* value < 0.001); the fourth-year resident doctors had the highest scores (mean = 9.24, SD = 1.6). As well, participants in the third and second years of postgraduate medical residency have a probability of adequate knowledge of ECG interpretation more than participants in the first year of residency (OR = 2.1, *p* value = 0.001) and (OR = 1.88, *p* value = 0.002), respectively.

**Conclusion:**

According to our research findings, resident doctors in departments of internal medicine and emergency medicine in Arabic nations have adequate ECG interpretation abilities; nevertheless, additional development is required to avoid misconceptions about critical cardiac conditions.

## 1. Introduction

Electrocardiography (ECG) is considered a crucial diagnostic tool in detecting the cardiovascular disorders, and it is the most frequently used tool among cardiology physicians, as 200 million electrocardiograms (ECGs) are performed annually. The ECG graph evaluates the heart rate and rhythm by recording the myocardial electrical activity *via* 12 external electrodes placed on the limbs and chest. Each electrode views the heart from one specific window to form the common ECG graph (1, 2). Early detection of severe cardiac abnormalities by academic interpretation of ECGs by health-care providers is essential, since cardiac conditions are very common and could be fatal (3).

Although an ECG is simple, cheap, portable, and easy to access, (2) interpreting the ECG is a challenging and complicated mission, and any mistake in the ECG interpretation could lead to undesirable outcomes, due to almost all cardiac disorders being considered as urgent consequences, as well as being the leading cause of death worldwide for both genders (4).

Electrocardiograms help the physicians, health-care providers, or clinicians to detect a wide range of life-threatening conditions, such as myocardial infarction, arrhythmias (atrial fibrillation/flutter, ventricular tachycardia/fibrillation), electrolyte imbalance, and some drugs’ toxicity. Accordingly, it is necessary for every doctor at emergency departments or intensive-care units to be able to define the risk signals on the ECG or detect the primary diagnosis as soon depending on the viewed graph on the ECG, which is classified as the most important diagnostic procedures when facing dangerous cases (4).

An accurate ECG interpretation is fundamental to providing high-quality patient care in several medical specialties, such as internal medicine and emergency medicine. Despite this, many studies have demonstrated that many resident doctors do not receive adequate training to develop their ECG interpretation skills (5). In spite of the global use of electrocardiograms (ECGs) as a diagnostic tool, ECG interpretation is linked to large mistake rates, particularly among physicians, general practitioners, and resident doctors (6). Studies showed that in developing and low-income countries, a prehospital ECG is considered a cost-efficient and worthwhile strategy for patients presenting with acute chest pains (7). Fortunately, many low-cost ECG machines are available within easy reach in low-income countries (8).

When dealing with urgent instances, such as myocardial infarction, finding a person who is competent to properly read the patient’s ECGs may be time-consuming and delay treatment. Since internal medicine and emergency medicine residents are the first health-care professionals inside hospitals to deal with such urgent cases, training to enhance their ECG interpretation abilities is crucial and might reduce mistake rates (9, 10). After checking the literature to define the studies that analyze the level of knowledge of Arabic resident doctors of ECG reading academically, we did not find any multinational study for this aim, so we conducted this study to assess the competency in electrocardiographic interpretation among emergency medicine and Internal medicine residents across Arab countries by emphasizing the most important abnormalities for the purpose of quality improvement and mitigating harm in emergency situations.

## 2. Methods

### 2.1. Study design and setting

An online cross-sectional study was conducted between October 7, 2022 and October 21, 2022 in eight Arabic countries (Syria, Jordan, Iraq, Qatar, Yemen, Egypt, Sudan, and Algeria). The study’s inclusion criteria were Arab resident doctors who underwent postgraduate training in internal medicine or emergency medicine departments from their first to fifth year. Resident doctors from other specialties and uncompleted surveys were excluded from the study. All participants were recruited into the study voluntarily without any pressure or coercion and were informed about the research group’s identity, their right to leave the study whenever they liked, their right to privacy and confidentiality, and the fact that only completed submissions would be analyzed.

This survey was adapted from a previously published study that involved a validated scale (11). We collected data from participants using convenience and snowball tactics. First, we translated the questionnaire from English into Arabic, and we guaranteed that all medical terminologies were translated based on the *Unified Medical Dictionary*. Second, we designed a google form questionnaire and sent it to participants through social media platforms by data collectors who informed the participants about the purpose of the study and indicated that it was not obligatory to participate in the study.

Data collectors visited hospitals to distribute the survey among potential respondents, and they were under daily supervision by the supervisors and the team leader. The minimal sample size was found by applying a single proportion of the population formula [*n* = [(Za/2)2P(1-P)]/d2]. With a 95% confidence level (Z a/2 = 1.96), a 5% margin of error, P = the proportion of emergency department internal medicine and emergency medicine residents who were competent in electrocardiogram interpretation (50%) and adding 5% for a non-response rate, 385 resident doctors were required to establish this study. The final size of the sample was 660 residents.

### 2.2. Measures

The questionnaire used to conduct this study has two sections. The first section involved the sociodemographic data, and the second section contained 12 clinical case questions with ECG records of the most significant ECG anomalies. The researchers formulated the survey from textbooks (11–13), published papers (14, 15), and clinical experience.

Section 2 of the survey primarily has two theoretical and 14 clinical questions. The final version was reduced to a total of 12 questions, and each question has four possible answers, of which only one is correct. To exclude the possibility of choosing the right answer by chance and to reduce the bias, one of the four answers was “I do not know.” Each respondent got one point for every correct answer, with a maximum score of 12 points.

After completing the questions, we changed the maximum score from 12 points to 10 points to make it easier to interpret the results. Respondents who scored 7.5/10.0 or more were considered competent in ECG interpretation; therefore, any respondent with a score less than 7.5 points assumed they had not reached the minimum level of theoretical proficiency in ECG interpretation.

#### 2.2.1. Sociodemographic variables

This section of the survey contained eight questions about the sociodemographic features of the study population. They ranged from questions about age, gender, hospital name, and years worked in the emergency department, whether the nurse had taken an ECG course, to three questions related to the course (type, duration of the course, and years since taking the course).

#### 2.2.2. Assessing ECG interpretation skills

In this section of the survey, we asked residents 12 questions, two theoretical questions, and 10 clinical case-related questions. The first two questions assess the participant’s knowledge of the order of ECG waves and intervals and their understanding of P wave. Then there were 10 questions covering diverse clinical scenarios with ECG records to determine the participant’s judgment and skills in interpreting various forms of ECGs. These records include atrial flutter, ventricular fibrillation, atrial fibrillation, pathological Q wave, atrioventricular third-degree bundle branch block, ventricular tachycardia, acute myocardial infarction, normal ECG, extra-ventricular systole, and atrial tachycardia.

### 2.3. Study validity and reliability

After evaluating the clarity of the questions by sending an online survey to 25 resident doctors and fixing the mistakes based on the comments we got, the reliability of the utilized scale (12 questions) was assessed using Interclass Correlation Coefficient on a small sample of 26 randomly chosen internal medicine and emergency residents. We calculated Cronbach’s alpha, which was 0.68, and the value of Cronbach’s alpha above 0.7 was defined to indicate adequate reliability (16). As a result, we indicated a somewhat satisfactory internal consistency.

### 2.4. Ethical consideration

This study was undertaken after the approval of the Syrian Ethical Society for Scientific Research (AS:2819B). Moreover, at least one ethical approval was taken from each country in our study. Respondents received a URL to access Google’s online survey and were asked on the first page of the survey if they consented to complete the survey. They were assured that the collected information would be used only for research purposes. Confidentiality and anonymity were respected in all steps of the study, and all answers were saved in an online protected database.

### 2.5. Statistical analysis

The statistical data analysis was performed using the STATA and Excel Microsoft programs. Categorical variables on sociodemographic characteristics were expressed using descriptive statistics and frequencies. We also categorized the knowledge levels into adequate and inadequate based on two modified cutoff points: above 75% and under 75% of the total score, respectively. A Test de Kruskal-Wallis was performed to determine the statistical difference in knowledge toward ECG interruption between the subgroups.

We conducted binary logistic regression to predict the possibility of the participants having adequate levels of ECG reading, depending on the other variables, including age, gender, specialty, training year, attending a previous ECG course, years since taking the course, type of course, and duration of the course. A value of *p* less than 0.05 was considered for statistical significance.

## 3. Results

### 3.1. Sociodemographic description of the study population

A total of 660 residents participated in the research. More than half (52.4%) were males, and the majority were internal medicine resident physicians. Only 36.2% of the participants have taken an ECG course. Most of these courses were less than 10 h long (22.7%), and 20.8% were held within the last 2 years. Participants were mostly urban (88.1%), and (88.1%) were employed in urban hospitals (Table 1).

**Table 1:** Socio-demographic features of the study participant.

Sociodemographic description of the study population (*n* = 660)		
	Frequency	Percentage
Age (years) [mean (SD)]	26.85	1.74
Gender		
Female	300	47.6%
Male	330	52.4%
Country		
Syria	222	35.2%
Yemen	86	13.7%
Egypt	109	17.3%
Jordan	94	14.9
Sudan	51	8.1%
Iraq	47	7.5%
Algeria	3	0.5%
Qatar	18	2.9%
Specialty		
Emergency medicine	100	15.9%
Internal medicine	530	84.1%
Training Year		
First	334	53.0%
Second	169	26.8%
Third	59	9.4%
Fourth	37	5.9%
Fifth	31	4.9%
Type of course		
Online	77	12.2%
Face-to-face	131	20.8%
Hybrid	20	3.2%
Has attended an ECG course		
Yes	228	36.2%
No	402	63.8%
Years since taking the course		
< 2 years	131	20.8%
2–5 years	63	10%
> 5 years	34	5.4%
Duration of course		
10–20 h	59	9.4%
> 20 h	26	4.1%
< 10 h	143	22.7%
Social status		
Single	462	73.3%
Married	168	26.7%
Origin		
Urban	506	76.6%
Rural	124	23.3%
Chronic disease		
Yes	79	12.5%
No	581	87.5%
Hospital site		
Urban	560	88.9%
Rural	70	11.1%

### 3.2. ECG interpretation

**Figure 1:**
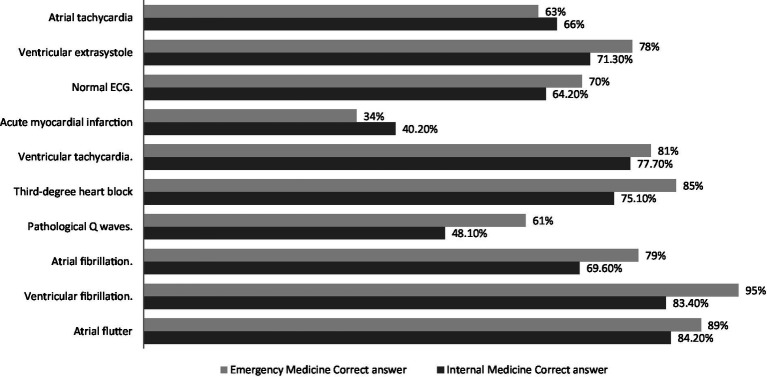
Electrocardiography (ECG) interpretation results.

Table 2 provides the correct and incorrect answers for the ECG interpretation scale. The ECG test waves have been properly arranged by 85.6% of the participants. Regarding atrial flutter, 84.9% of the participants provided correct responses, whereas 14.8% reported wrong answers for ventricular fibrillation. Less than three-quarters of the participants correctly identified the atrial fibrillation cases (71.1%), while approximately half of the participants failed to recognize the pathological Q wave (49.8%). As for ventricular tachycardia, 78.3% of the participants correctly identified the ventricular tachycardia case, while only 39.2% of the participants correctly diagnosed the acute myocardial infarction case. More than half of the participants (65.1%) correctly recognized a normal ECG, whereas 72.4% properly diagnosed ventricular extra-systole. The atrial tachycardia condition was misdiagnosed by 34.4% of the subjects (Table 2) and (Figure 1).

**Table 2:** ECG interpretation results.

ECG interpretation results	Frequency	Percentage
1. What is the correct order of ECG waves and intervals?		
A. T wave, P wave, QRS complex, PR interval, ST interval, U wave.		
B. P wave, QRS complex, T wave, PR interval, ST interval, U wave.		
C. QRS complex, P wave, PR interval, T wave, ST interval, U wave.		
D. I do not know.		
Correct	539	85.6%
False	91	14.4%
2. If in an ECG the P wave does not appear, what is your first thought?		
A. There is a conduction problem between the ventricles.		
B. There is a conduction problem between the atriums.		
C. It is normal; it does not have to appear in an ECG.		
D. I do not know.		
Correct	542	86%
False	88	14%
3. You perform an ECG and observe this register. What do you think it might be?		
A. A third-degree heart block		
B. An atrial flutter		
C. A supraventricular tachycardia		
D. I do not know.		
Correct	535	84.9%
False	95	15.1%
4. You perform an ECG and observe this register. How would you act?		
A. Ask for help without leaving the patient alone because it is a ventricular fibrillation.		
B. Ask for help without leaving the patient alone because it is an atrial fibrillation.		
C. Perform another ECG because it looks like there may be interference.		
D. You do not know how to act but you know it must be a serious problem.		
Correct	537	85.2%
False	93	14.8%
5. A patient comes to the emergency department because of respiratory distress. He has 140 beats per minute. You perform an ECG and observe the following:		
A. It is an atrial tachycardia.		
B. It is an atrial fibrillation.		
C. It is an atrial extra systole.		
D. I do not know.		
Correct	448	71.1%
False	182	28.9%
6. A patient comes to the emergency department with precordial pain for more than 8 h. You perform a 12-branch ECG. After observing the ECG, what catches your attention?		
A. You can see pathological pauses.		
B. You can see pathological Q waves.		
C. The patient has a low cardiac rhythm.		
D. I do not know.		
Correct	316	50.2%
False	314	49.8%
7. What pathology do you think the patient has as demonstrated on this ECG?		
A. A first-degree heart block		
B. He does not have any pathology		
C. A third-degree heart block		
D. I do not know.		
Correct	483	76.7%
False	147	23.3%
8. A hospitalized patient who had had surgery because of an AMI is transferred to the emergency department to be monitored because his vital signs are unstable. You perform an ECG and observe the following:		
A. The patient presents with a ventricular tachycardia.		
B. The patient presents with a supraventricular tachycardia.		
C. The patient presents with an atrial tachycardia		
D. I do not know		
Correct	493	78.3%
False	137	21.7%
9. You are in triage and call a patient who reports medium-intensity precordial pain. He tells you that the pain appeared after leaving an important meeting 2 h ago. He is 52 years of age and hypertensive; a few months ago he was diagnosed with type 2 diabetes mellitus. You perform a 12-branch ECG and observe the following:		
A. It is a supraventricular tachycardia.		
B. It is an acute myocardial infarction		
C. It is an acute myocardial infarction with a pathological Q wave.		
D. I do not know.		
Correct	247	39.2%
False	383	60.8%
10. A 24-year-old athletic, slim man comes to the emergency department. He reports feeling a pricking sensation in the left area of his chest since he finished exercise 3 h earlier. You perform an ECG and observe the following:		
A. It is an atrial bradycardia.		
B. He has conduction problems.		
C. It is a normal ECG.		
D. I do not know.		
Correct	410	65.1%
False	220	34.9%
11. A patient with digitalis intoxication comes from a hospitalization ward. Before monitoring him, you perform an ECG and observe the following:		
A. You observe an atrial extra systole		
B. You observe a ventricular extra systole		
C. You observe that he has a pacemaker.		
D. I do not know.		
Correct	456	72.4%
False	174	27.6%
12. A 30-year-old woman comes to the emergency department reporting palpitations, chest tightness, and dyspnea. You perform an ECG and observe the following:		
A. It is a ventricular tachycardia.		
B. It is an atrial extra systole.		
C. It is an atrial tachycardia		
D. I do not know.		
Correct	413	65.6%
False	217	34.4%

### 3.3. Sample description of hospital, years of work experience, and ECG training

Only three variables have a significant difference in the score, including gender, social status, and duration of the training course. Male participants scored higher (8.79 ± 2.32) than females, while participants who attended courses with more than 20 training hours scored higher than courses with fewer training hours duration (8.65 ± 2.58), see Table 3.

**Table 3:** Sample description of hospital, years of work experience, and ECG training.

Sample description of hospital, years of work experience, and ECG training		
Variables	Mean/SD	*p* value
Gender		0.03
Male	8.79/2.32	
Female	8.39/2.41	
Social status		0.048
Single	8.49/2.41	
Married	8.91/2.37	
Has received training?		0.97
Yes	8.60/2.28	
No	8.60/2.42	
Time since receiving training		0.38
2–5 years	8.71/2.50	
> 5 years	7.94/2.50	
< 1 year	8.71/2.42	
Specialty		0.2
Emergency medicine	8.88/2.10	
Internal medicine	8.55/2.41	
Type of training		0.99
Online	8.65/2.06	
Hybrid	8.45/3.20	
Face-to-face	8.89/2.26	
Hospital site		0.91
Urban hospital	8.61/2.37	
Rural hospital	8.57/2.4	
Duration of the training		0.02
< 10 h	8.57/2.28	
10–20 h	8.64/2.20	
> 20 h	8.65/2.58	

### 3.4. Prediction of the ECG interpretation

Logistic regression was used in Table 4 to determine the appropriate level of knowledge regarding ECG interpretation. Training year was the only variable significantly associated with ECG interpretation, in which participants in their third year of residency have a higher probability of correct ECG interpretation than participants in their first year of residency (OR = 2.15, *p* value < 0.05), see Table 4.

**Table 4:** Binary logistic regression to determine the appropriate level of knowledge regarding ECG interpretation.

Variable	Adjusted				Un-adjusted			
	*p* value	OR	95% C.I		*p* value	OR	95% C.I	
			Lower	Upper			Lower	Upper
Age	0.254	1.068	0.954	1.195	0.002	1.153	1.052	1.264
Gender (Male: Ref)	1							
Female	0.298	0.840	0.606	1.166	0.236	0.828	0.605	1.132
Specialty (Emergency medicine: Ref)	1							
Internal medicine	0.726	0.920	0.578	1.464	0.229	0.768	0.500	1.181
Training year (first year: Ref)	1							
Second year	0.002	1.881	1.267	2.793	0.000	1.984	1.362	2.889
Third year	0.016	2.154	1.157	4.008	0.002	2.477	1.393	4.404
Fourth year	0.106	1.921	0.870	4.240	0.048	2.008	1.006	4.008
Fifth year	0.508	1.315	0.585	2.960	0.178	1.662	0.793	3.483
Attending a previous ECG course (Yes: Ref)	1							
No	0.777	0.831	0.231	2.991	0.751	1.054	0.762	1.459
Years since taking the course (< 2 years: Ref)	1				0.940	1.015	0.684	1.506
2–5 years	0.161	1.800	0.791	4.096	0.293	1.333	0.780	2.279
>5 years	0.087	2.170	0.892	5.278	0.511	0.789	0.390	1.597
Type of course (Online: Ref)	1				0.917	0.974	0.598	1.587
Face-to-face	0.620	0.763	0.262	2.222	0.820	1.047	0.706	1.553
Hybrid	0.700	0.820	0.299	2.248	0.385	1.500	0.600	3.748
Duration of course (10–20 h: Ref)	1				0.943	0.986	0.673	1.444
>20 h	0.546	0.756	0.305	1.874	0.715	1.107	0.641	1.913
<10 h	0.758	0.858	0.324	2.276	0.449	1.364	0.611	3.042

## 4. Discussion

The ability of clinicians to interpret ECGs accurately determines the results for their patients. This study was carried out with the intention of evaluating the level of competency in ECG interpretation made by emergency medicine and internal medicine residents across the Arab countries.

Particular attention was paid to the most significant abnormalities for the purposes of enhancing the level of care provided and reducing the risk of injury in the event of an emergency. The internal medicine and emergency medicine house residents who participated in this study only managed to attain a limited overall competency score. According to the findings of other research, physicians in training have a 36–80% accuracy rate when it comes to identifying ECG diagnoses (17–24).

We discovered that there was not a significant difference between emergency medicine residents’ and internal medicine residents’ perceptions of how adequately competent they were with ECG. Although the overall performance was low for both fields of study (rates of incorrect diagnosis were 58% for complete heart block and 8% for myocardial infarction), emergency medicine residents have similar skill levels in ECG interpretation compared to medicine residents. This could be due to the equal amount of expertise within both emergency medicine and internal medicine residents. This was found in a study conducted in New York (25). On the ECG of ventricular tachycardia, we discovered that emergency medicine residents had better scores. In fact, ventricular tachycardia is an emergency condition, where more likely emergency medicine residents will be exposed to this condition indicating more accurate interpretation of ventricular tacycardia on an ECG. In contrast, the research conducted in New York revealed that internal medicine residents had much higher scores on the ECG of ventricular tachycardia. In addition, findings from similar studies indicate that cardiologists may do better than other physicians. This could be particularly accurate when there is a lack of precise clinical data of the patients with severe conditions (26).

Compared to participants in their first year of residency, individuals in their third year of residency have a higher likelihood of making the correct ECG interpretation. Senior residents were more likely than junior residents to report ECGs on their own, according to a Nigerian study (27), which compared the two groups. As a result of their exposure to and expertise with interpreting ECGs during their residency, higher level residents are more likely to interpret ECGs accurately. In addition, our findings revealed that female participants performed worse than their male colleagues, which it might be claimed that men doctors are better able to handle severe cardiovascular diseases than women doctors since the ECG is a critical diagnostic tool that requires precision and care when investigating these cases like heart failure and ventricular fibrillation. Nevertheless, another study revealed that fatality rates for both women and men were lower in emergency departments when the treating physician was a woman because female doctors tend to listen more to their patients (28).

There have been a few studies in the past that have demonstrated how training may increase one’s ability to read ECGs. Of a total of eight ECGs, Hatala et al. examined the responses of 30 fourth-year medical students, 15 residents in internal medicine, and 15 cardiologists. They showed significant improvement at each level of training (22). There are some patterns on an ECG that signify problems that a resident may be required to treat urgently. Acute myocardial infarction, complete heart block, ventricular fibrillation, and ventricular tachycardia are some of the conditions that fall under this category.

In our research, we looked at three different types of electrocardiographic emergencies: acute myocardial infarction, ventricular tachycardia, and complete heart block. Of these, 60.8% of patients had the wrong diagnosis for acute myocardial infarction, 21.7% of patients were misdiagnosed with ventricular tachycardia, and 23.3% had misdiagnosed complete heart block. In contrast, the New York research found that ST segment elevation myocardial infarction was incorrectly diagnosed in 8% of patients, ventricular tachycardia was incorrectly identified in 11% of patients, and complete heart block was incorrectly diagnosed in 58% of patients. In many cases, these diagnoses call for immediate medical intervention, and getting them wrong might have huge consequences. As a result, steps should be taken to empower residents in terms of ECG interpretation, such as holding standard ECG interpretation courses and techniques to strengthen ECG interpretation training and learning, such as ECG display training packages. Participants’ lack of proficiency in interpreting ECGs can be improved further by utilizing computer-aided diagnosis and a focus on medical imaging.

There are a variety of measures that might be taken in order to improve ECG expertise. It has been demonstrated that even brief training in ECG interpretation may considerably improve a person’s ability to read electrocardiograms (17). The majority of the time, these diagnoses require prompt medical care, and getting them incorrect might have extremely serious implications. Improving one’s knowledge of ECG may be accomplished through a range of different approaches that can be pursued. It has been established that even a cursory instruction in the interpretation of electrocardiograms may significantly increase a person’s ability to read electrocardiograms (20, 29).

The American Boards of Internal Medicine and Emergency Medicine have mandated that all staff members must undergo ECG training, undergo an initial evaluation of their level of proficiency, and continue to demonstrate that they can maintain their level of competency over time. It was suggested by Salerne et al. that the determination of initial competency in ECG interpretation at the end of residency training should be based on periodic objective assessment and documentation of resident interpretation skills in a clinical context rather than the completion of a minimum number of interpretations. This was in contrast to the traditional method of determining initial competency in ECG interpretation, which was based on the completion of a minimum number of interpretations. In contrast to the conventional approach, which consisted of basing initial competency on the successful completion of a certain number of interpretations (26).

### 4.1. Strengths and limitations

When attempting to make sense of the findings of our research, there are a few limitations that need to be noted. To begin, there were a far lower number of emergency medicine residents than there were internal medicine residents. It is possible that this is the reason why there is not a substantial association between the postgraduate year and competency. Second, for the purposes of the study, an ECG sample that was both small and somewhat arbitrary was selected. Even while we assume that these ECGs accurately reflected the majority of patients’ conditions, it is likely that the residents’ findings might have been different if they had been given other ECGs.

This study used both an online format with an accompanying online questionnaire as well as an in-person interview. Due to the fact that the test is administered online, locals have the ability to look the answers up, which can result in inaccurate scoring. When conducting the survey, using a questionnaire based on an in-person interview helped to lessen the probability that respondents relied on information obtained from other sources when answering the survey questions. This is a strength of the approach. One of the other strengths of this study is that it provides a clinical scenario alongside each ECG, which has a positive impact on the interpretation. Participants in our study were under no obligation to take part, and they were not threatened or coerced in any way by potential outcomes should they choose not to take part.

## 5. Conclusion

We have uncovered inherent drawbacks in the interpretation of ECGs, which may have major consequences for the medical treatment provided to residents and patients. As heart conditions are quite frequent and potentially result in death, it is crucial for medical professionals to notice and evaluate any abnormalities on an ECG as soon as possible. Extra training is required, especially in the treatment of cardiac crises. The primary focus of research to come should be on developing and evaluating effective methods for ECG interpretation expertise.

### Data collection group

The data collection group has contributed equally to collect the responses by sharing the online google form survey to the doctors at the departments of internal medicine and emergency medicine.

1. Diaa Yousef: Faculty of Medicine, Aleppo University, Aleppo, Syria (dr.diaa997@gmail.com)
2. Muhammad Taib: Faculty of Medicine, Damascus University, Damascus, Syria (mohamedtayeeb7@gmail.com)
3. Yomen Alabrash: Faculty of Medicine, Albaath University, Homs, Syria (yomenstar2000@gmail.com)
4. Tarek Mansour: Faculty of Medicine, Damascus University, Damascus, Syria (Mansourt300@gmail.com)
5. Lujain Al Shal: Faculty of Medicine, Damascus University, Damascus, Syria (alshallujain@gmail.com)
6. Noor Tayeb: Faculty of Medicine, Albaath University, Homs, Syria (noortb553@gmail.com)
7. Hana Mousa: Faculty of Medicine, Damascus University, Damascus, Syria (hanna1761998@gmail.com)
8. Wehba Hraiz: Faculty of Medicine, Damascus University, Damascus, Syria (Hraizwehba@gmail.com)
9. Mahmoud Hasan Kallih: Faculty of Medicine, Tishreen University, Lattakia, Syria (mahmoudkallih@gmail.com)
10. Lana Sheet: Faculty of Medicine, Aleppo University, Aleppo, Syria (Lanasheitt3@gmail.com)
11. Nour AL Salama: Faculty of Medicine, Damascus University, Damascus, Syria (Nour.alsalama123@gmail.com)
12. Mohamad Shaban: Faculty of Medicine, Damascus University, Damascus, Syria (mohamadshapaan3@gmail.com)
13. Ranim Joumaa: Faculty of Medicine, Damascus University, Damascus, Syria (rnymjmtt@gmail.com)
14. Zahra Odeh: Faculty of Medicine, Damascus University, Damascus, Syria (Zahorazoza4@gmail.com)
15. Kinda Almanla: Faculty of Medicine, Aleppo University, Aleppo, Syria (Kinda77ibrahim@gmail.com)
16. Nour Mezketli: Faculty of Medicine, Aleppo University, Aleppo, Syria (nour.mzk1@gmail.com)
17. Deena Mufead Nafea: Faculty of Medicine, University of Jordan, Amman, Jordan (Deena.nafea98@gmail.com)
18. Eman Alrefai: Faculty of Medicine, Yarmouk University, Irbid, Jordan (Emanrefai11@gmail.com)
19. Fatima Alkubaisi: Faculty of Medicine, University of Jordan, Amman, Jordan (Fatima_alkubaisi@yahoo.com)
20. Omar Wafi: Faculty of Medicine, Jordan University of Science and Technology, Amman, Jordan (o.s.w.wafi@gmail.com)
21. Waheeb Ali Ahmed Al-Garadi: Faculty of Medicine, Thamar University, Yemen (Waheeb99944@gmail.com)
22. Hadeel Alsharjabi: Faculty of Medicine, Sana’a University, Yemen (hadeelfuadalsharjabi89@gmail.com)
23. Lina Muneer Mohammed Al-Qalisi: Faculty of Medicine, Sana’a University, Yemen (linamonir1@gmail.com)
24. Qasim Jamal Qasim Al-dhaheri: Faculty of Medicine, 21 september university, Yemen (Qasimjamal53@gmail.com)
25. Thoria Hassan Kaid Basha: Faculty of Medicine, Sana’a University, Yemen (thoria20@yahoo.com)
26. Hiam Al-Atnah: Faculty of Medicine, Emirates International University, Yemen (hiam.alatnah@gmail.com)
27. Bushra Al Mkhlafi: Faculty Medicine, Sanaa University, Yemen
28. Maysa Ahmed Hamoud Al-khairy: Faculty of Medicine, Sana’a University, Yemen (just.mam47@gmail.com)
29. Heba Mansour: Faculty of Medicine, Sana’a University, Yemen (hebhkhaled7@gmail.com)
30. Heba Hamouda: Faculty of Medicine, Menoufia University, Egypt (hebahamouda53@med.menofia.edu.eg)
31. Nour Kamsheh: Faculty of Medicine, Misr University for Science and Technology, Egypt (nourkamsheh@gmail.com)
32. Hadeer Hafez: Faculty of Medicine, October 6^th^ University, Egypt (Hadeerhafez0@gmail.com)
33. Donia Farhat: Faculty of Medicine, Tanta University, Egypt (donia_farhat@yahoo.com)
34. Aiman Ahmad Al-Touny: Faculty of Medicine, Suez Canal University, Egypt (aimantouny@yahoo.com)
35. Ayman Hussen: Faculty of Medicine, Alexandria University, Alexandria, Egypt (ayman.hussen122@gmail.com)
36. Zaid Mohammed: Faculty of Medicine, Al Neelain University, Khartoum, Sudan (zaidaaed@gmail.com)
37. Mohammed Ahmed Salah Mohammed Ahmed Elgak: Faculty of Medicine and Health Science, Kassala University, Sudan (Mohammedahmed6218@gmail.com)
38. Hasan Ahmed Battikh: Faculty of Medicine, Al Neelain University, Khartoum, Sudan (Hasanlord9@gmail.com)
39. Mohamed Idries Mohamed Idries: Faculty of Medicine, Omdurman Islamic University, Sudan (mohammededris1997129@gmail.com)
40. Fatima Elsamani: Faculty of Medicine, University of Jezira, Sudan (Fatimaalsamani20@gmail.com)
41. Maab Magmoud Mohamed Attaelmnan: Faculty of Medicine, University of Jezira, Sudan (maabm29@gmail.com)
42. Mohamed Amir: Faculty of Medicine, University of Algiers, Algeria (raismohammedamir@gmail.com)
43. Mawahib Hajhamed: Faculty of Medicine, Ahfad University for Women, Omdurman, Suda.

## Data availability statement

The datasets presented in this study can be found in online repositories. The names of the repository/repositories and accession number(s) can be found in the article/Supplementary material.

## Ethics statement

This study was undertaken after the approval of the Syrian Ethical Society for Scientific Research (AS:2819B). Moreover, at least on ethical approval was taken from each inquired country in our study.

## Author contributions

AR and SS: conceptualization, methodology, formal analysis, writing-original draft, and review and editing. SS, HA, HB, YA, KA, YK, BS, BR, MA, IA-F, WH, MR, AG, and EE-S: conceptualization and writing the original draft. WH, AR, AG, and EE-S: proofreading and reviewing and editing the final draft of the manuscript. All authors contributed to the article and approved the submitted version.

## Conflict of interest

The authors declare that the research was conducted in the absence of any commercial or financial relationships that could be construed as a potential conflict of interest.

## Publisher’s note

All claims expressed in this article are solely those of the authors and do not necessarily represent those of their affiliated organizations, or those of the publisher, the editors and the reviewers. Any product that may be evaluated in this article, or claim that may be made by its manufacturer, is not guaranteed or endorsed by the publisher.
