# Supplementary material for: Assessment of the competence in electrocardiographic interpretation among Arabic resident doctors at the emergency medicine and internal medicine departments: A multi-center online cross-sectional study
Source: Front Med (Lausanne). 2023 Apr 24;10:1140806. doi: 10.3389/fmed.2023.1140806 (PMC10165895; doi:10.3389/fmed.2023.1140806)
Supplement: Supplementary file 1 [file Data_Sheet_1.docx]

Supplementary Materials

Supplementary material 1, questionnaire about ECG interpretation skills among Arab residents in emergency medicine and internal medicine departments:

Part 1 : Basic and demographic information

1. Are you willing to participate in this study?
2. Agree
3. Disagree
4. How old are you? ________ .
5. Are you:
6. Male
7. Female
8. What is your marital status?
9. Single
10. Married
11. Widowed
12. Divorced
13. Where do you live?
14. City
15. Countryside
16. Are you suffering from any chronic illness?
17. Yes
18. No
19. What is your specialty?
20. Emergency medicine
21. Internal medicine
22. You are at your ____ year of the residency program.
23. First
24. Second
25. Third
26. Fourth
27. Fifth
28. Sixth
29. The hospital in which you are working in is located in the:
30. City
31. Countryside
32. Have you ever taken an ECG course?
33. Yes
34. No

(If yes, continue the following questions. If no, move to part 2)

1. How many years have passed since taking the course?
2. < 2 years
3. 2-5 years
4. > 5 years
5. What is the type of the course?
6. Online
7. Face-to-face
8. Hybrid
9. How long did the course last?
10. < 10 hours
11. 10-20 hours
12. > 20 hours

Part 2 : Assessment of the ECG interpretation skills

1. What is the correct order of ECG waves and intervals?
2. P wave, QRS complex, T wave, PR interval, ST interval, U wave (correct answer)
3. T wave, P wave, QRS complex, PR interval, ST interval, U wave
4. QRS complex, P wave, PR interval, T wave, ST interval, U wave
5. I do not know.
6. If in an ECG the P wave does not appear, what is your first thought?
7. There is a conduction problem between the ventricles.
8. There is a conduction problem between the atriums (correct answer).
9. It is normal; it does not have to appear in an ECG.
10. I do not know.
11. You perform an ECG and observe this register.


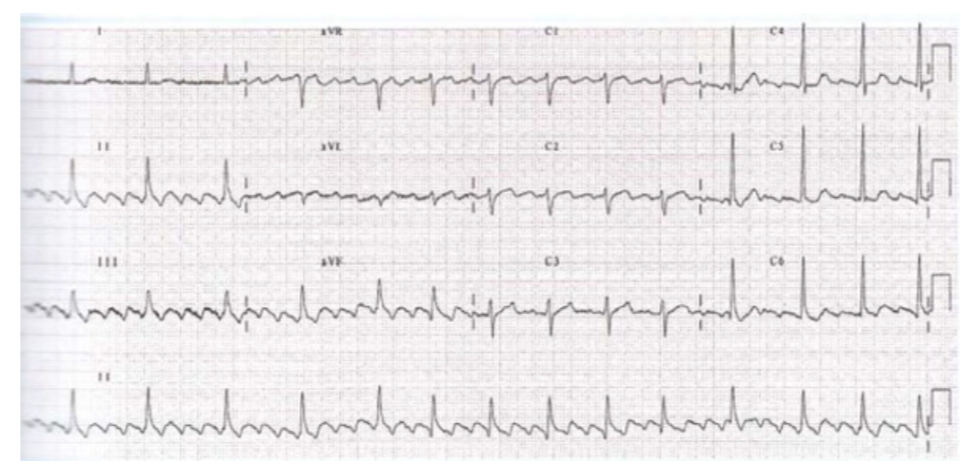


What do you think it might be?

1. A third-degree heart block
2. An atrial flutter (correct answer)
3. A supraventricular tachycardia
4. I do not know.
5. You perform an ECG and observe this register.
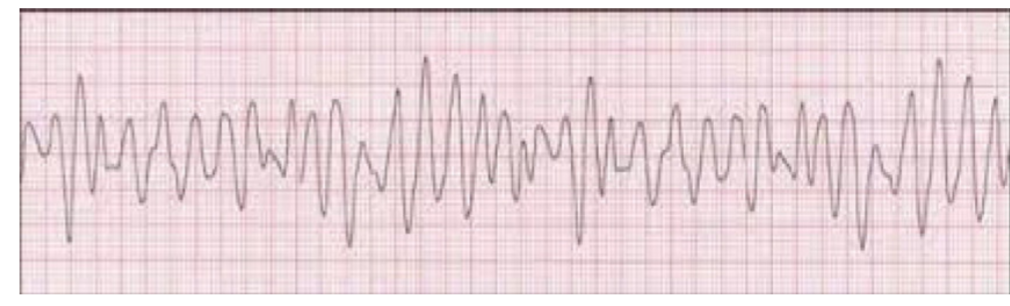


How would you act?

1. Ask for help without leaving the patient alone because it is a ventricular fibrillation (correct answer).
2. Ask for help without leaving the patient alone because it is an atrial fibrillation.
3. Perform another ECG because it looks like there may be interference
4. You do not know how to act but you know it must be a serious problem
5. A patient comes to the emergency department because of respiratory distress. He has 140 beats per minute. You perform an ECG and observe the following:
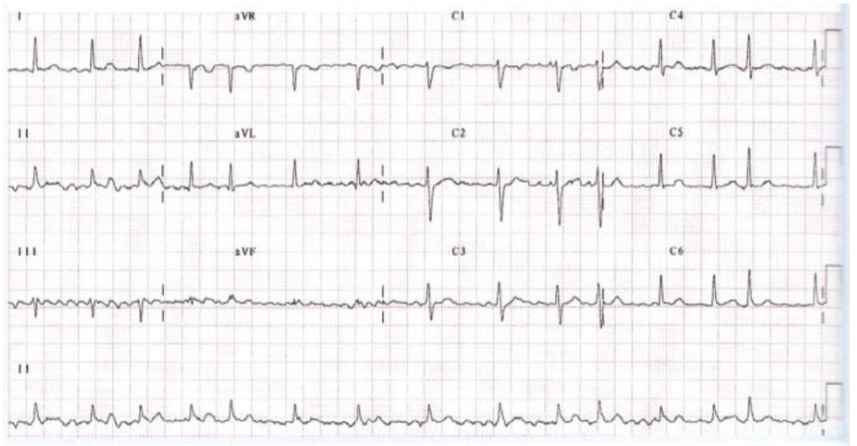

6. It is an atrial tachycardia.
7. It is an atrial fibrillation (correct answer).
8. It is an atrial extrasystole.
9. I do not know.
10. A patient comes to the emergency department with precordial pain for more than 8 hours. You perform a 12-branch ECG.After observing the ECG, what catches your attention?
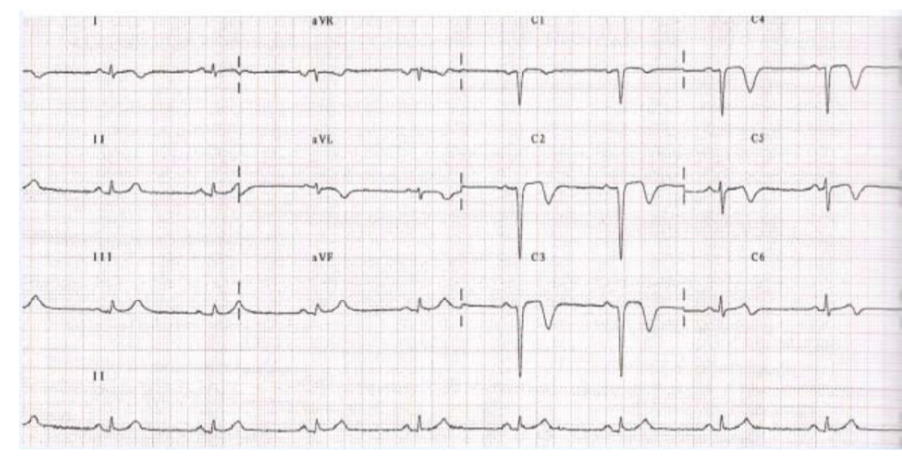

11. You can see pathological pauses.
12. You can see pathological Q waves (correct answer).
13. The patient has a low cardiac rhythm.
14. I do not know.
15. What pathology do you think the patient has as demonstrated on this ECG?
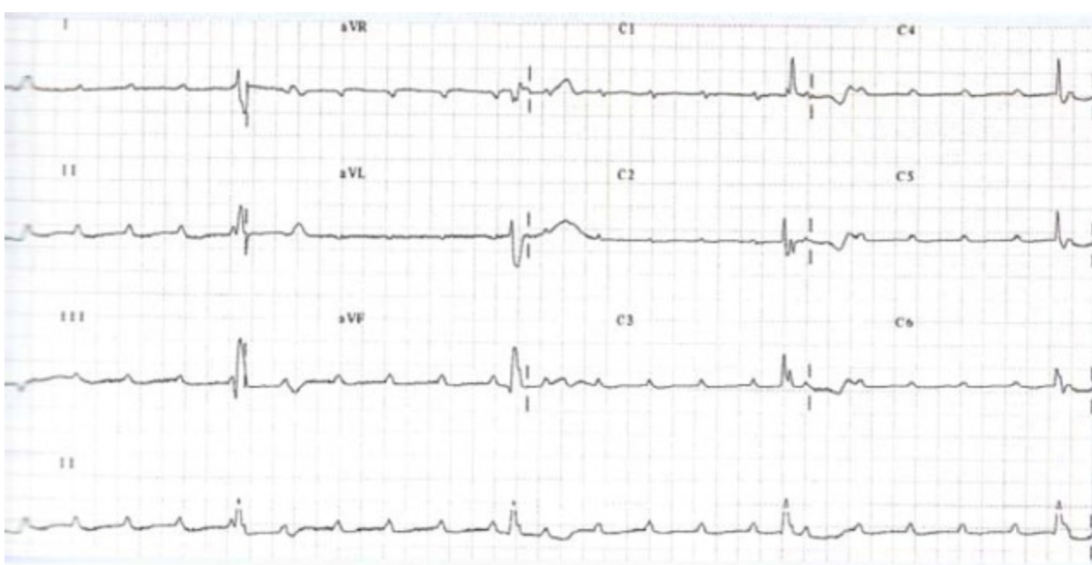

16. A first-degree heart block
17. He does not have any pathology.
18. A third-degree heart block (correct answer).
19. I do not know
20. A hospitalized patient who had had surgery because of an AMI is transferred to the emergency department to be monitored because his vital signs are unstable.
    You perform an ECG and observe the following:
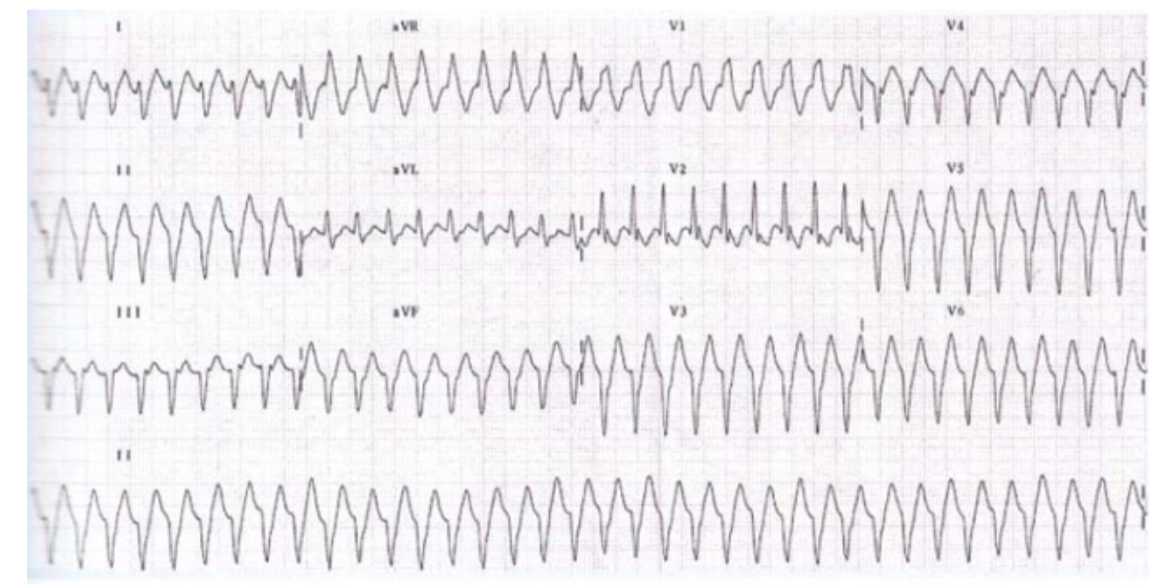

21. The patient presents with a ventricular tachycardia (correct answer).
22. The patient presents with a supraventricular tachycardia.
23. The patient presents with an atrial tachycardia.
24. I do not know.
25. You are in triage and call a patient who reports medium-intensity precordial pain.
    He tells you that the pain appeared after leaving an important meeting 2 hours ago. He is 52 years of age and hypertensive; a few months ago he was diagnosed with type 2 diabetes mellitus. You perform a 12-branch ECG and observe the following:
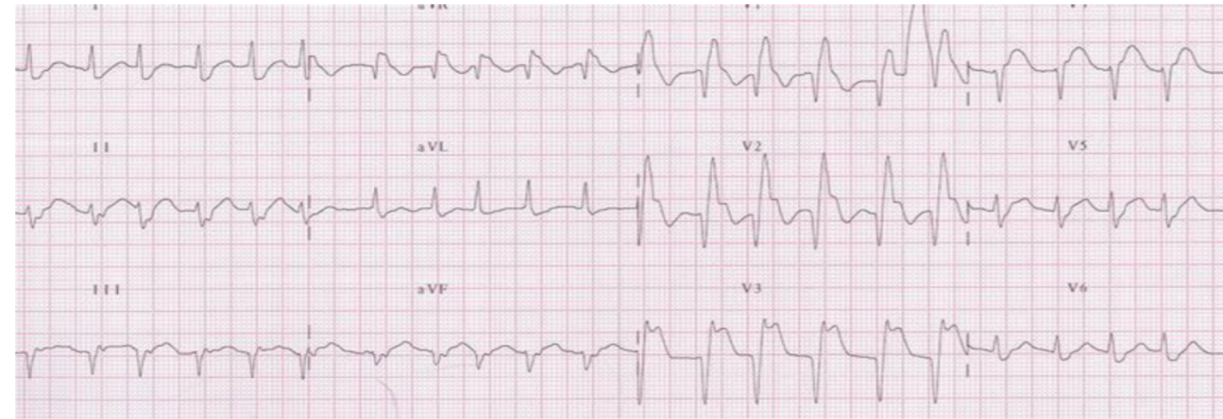

26. It is a supraventricular tachycardia.
27. It is an acute myocardial infarction (correct answer).
28. It is an acute myocardial infarction with a pathological Q wave.
29. I do not know.
30. A 24-year-old athletic, slim man comes to the emergency department.
    He reports feeling a pricking sensation in the left area of his chest since he finished exercise 3 hours earlier. You perform an ECG and observe the following:
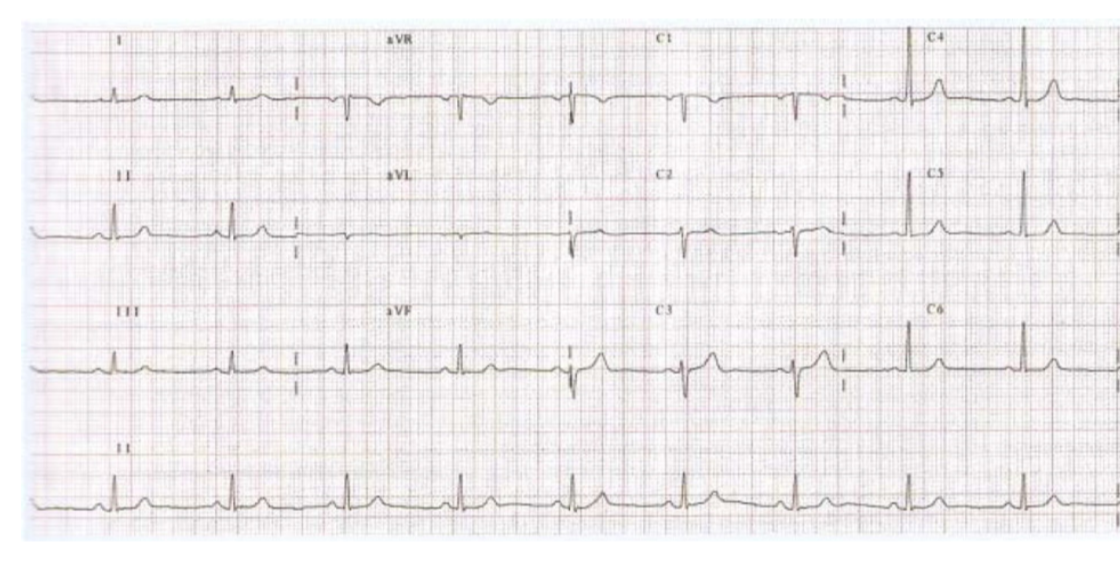

31. It is an atrial bradycardia
32. He has conduction problems.
33. It is a normal ECG (correct answer).
34. I do not know.
35. A patient with digitalis intoxication comes from a hospitalization ward.
    Before monitoring him, you perform an ECG and observe the following:
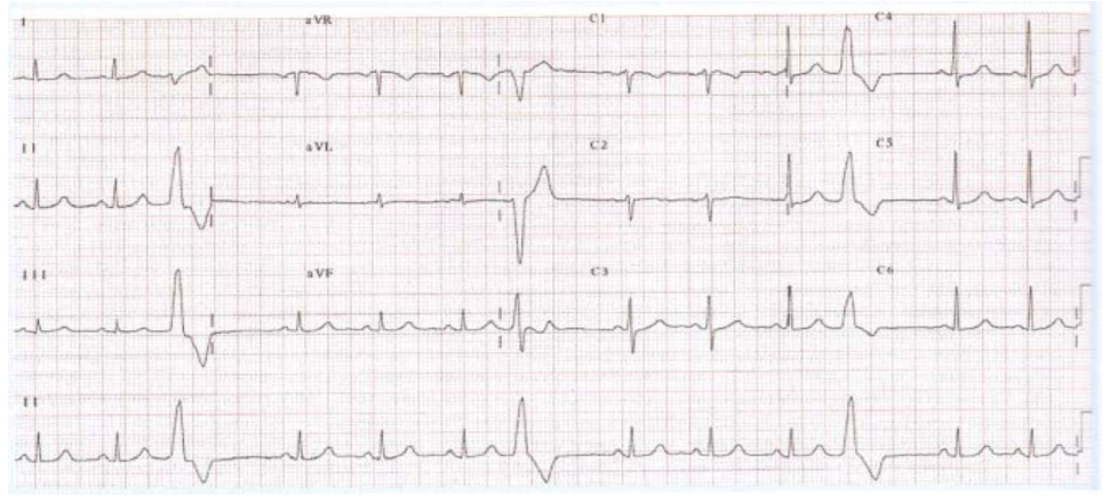

36. You observe an atrial extrasystole.
37. You observe a ventricular extrasystole (correct answer).
38. You observe that he has a pacemaker.
39. I do not know
40. A 30-year-old woman comes to the emergency department reporting palpitations, chest tightness, and dyspnea. You perform an ECG and observe the following:
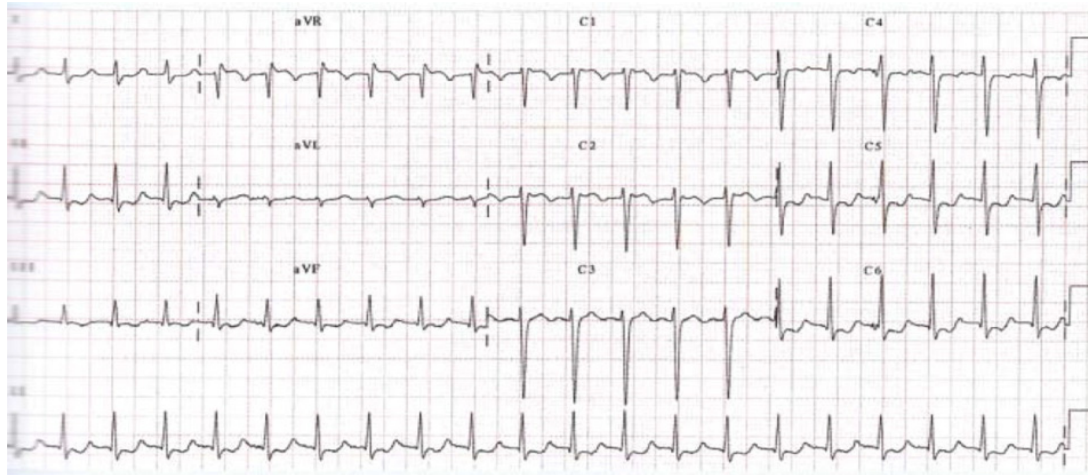

41. It is a ventricular tachycardia.
42. It is an atrial extrasystole.
43. It is an atrial tachycardia ( correct answer ).
44. I do not know.
